# Enhancing the discovery and development of immunotherapies for cancer using quantitative and systems pharmacology: Interleukin-12 as a case study

**DOI:** 10.1186/s40425-015-0069-x

**Published:** 2015-06-16

**Authors:** David J Klinke

**Affiliations:** Department of Chemical Engineering and Mary Babb Randolph Cancer Center, West Virginia University, Morgantown, WV 25606 USA

## Abstract

Recent clinical successes of immune checkpoint modulators have unleashed a wave of enthusiasm associated with cancer immunotherapy. However, this enthusiasm is dampened by persistent translational hurdles associated with cancer immunotherapy that mirror the broader pharmaceutical industry. Specifically, the challenges associated with drug discovery and development stem from an incomplete understanding of the biological mechanisms in humans that are targeted by a potential drug and the financial implications of clinical failures. Sustaining progress in expanding the clinical benefit provided by cancer immunotherapy requires reliably identifying new mechanisms of action. Along these lines, quantitative and systems pharmacology (QSP) has been proposed as a means to invigorate the drug discovery and development process. In this review, I discuss two central themes of QSP as applied in the context of cancer immunotherapy. The first theme focuses on a network-centric view of biology as a contrast to a “one-gene, one-receptor, one-mechanism” paradigm prevalent in contemporary drug discovery and development. This theme has been enabled by the advances in wet-lab capabilities to assay biological systems at increasing breadth and resolution. The second theme focuses on integrating mechanistic modeling and simulation with quantitative wet-lab studies. Drawing from recent QSP examples, large-scale mechanistic models that integrate phenotypic signaling-, cellular-, and tissue-level behaviors have the potential to lower many of the translational hurdles associated with cancer immunotherapy. These include prioritizing immunotherapies, developing mechanistic biomarkers that stratify patient populations and that reflect the underlying strength and dynamics of a protective host immune response, and facilitate explicit sharing of our understanding of the underlying biology using mechanistic models as vehicles for dialogue. However, creating such models require a modular approach that assumes that the biological networks remain similar in health and disease. As oncogenesis is associated with re-wiring of these biological networks, I also describe an approach that combines mechanistic modeling with quantitative wet-lab experiments to identify ways in which malignant cells alter these networks, using Interleukin-12 as an example. Collectively, QSP represents a new holistic approach that may have profound implications for how translational science is performed.

## Introduction

Following the early clinical observations of William B. Coley, harnessing the immune system to cure cancer has been difficult to achieve in the clinic. Recent FDA approval of immune checkpoint modulators for cancer has renewed enthusiasm in multiple communities. Patient groups are excited about the prospect for a cure. For patients with a historically poor prognosis, like metastatic melanoma, immune checkpoint modulators provide real hope for a favorable outcome following treatment. The translational cancer immunology community has been invigorated by the clinical affirmation of the conceptual approach. Researchers that may have felt marginalized during the oncogene era have emerged into the scientific spotlight based on the impressive clinical trial results [1]. The pharmaceutical industry, which has been plagued by clinical failures and a dismal return on investment [2, 3], is rushing into the space in order to carve out a therapeutic niche as they envision a dramatic change in the clinical landscape towards immunotherapies [4].

Despite these sources of enthusiasm, there are a number of persistent translational challenges associated with cancer immunotherapy [5]. These hurdles include the limits in the fidelity of animal models to predict efficacy of immunotherapies in humans and incomplete understanding of the dynamics of treatment response. Identifying the specific immune escape mechanisms present within a patient’s tumor presents a hurdle for broadening the subset of patients that receive clinical benefit. Moreover, these specific immune escape mechanisms can be heterogeneous, such that they can vary among tumor lesions within a patient and vary among tumor lesions within an anatomical location across a patient population. Financial hurdles associated with translating science into patients are a direct consequence of clinical failures, where the lack of biomarkers of immune response and objective response criteria that reflect the underlying response dynamics to immunotherapy present hurdles for demonstrating clinical efficacy. Collectively, the hurdles associated with bringing cancer immunotherapies to the clinic have financial implications even after FDA approval. For instance, Dendreon, which markets an immunotherapy for prostate cancer that has an estimated $93,000 price tag, recently filed for Chapter 11 bankruptcy citing underperforming sales and increased competition from other therapies for prostate cancer. Given the deserved enthusiasm for cancer immunotherapy, a growing concern in the field is how do we channel these energies and leverage decades of basic science research to sustain the incredible current successes [6]. In the following paragraphs, I will summarize some of the translational challenges facing cancer immunotherapy and provide an overview of some potential solutions that leverage a more contemporary view of disease pathophysiology and both computational and experimental developments.

## Review

### Contemporary drug discovery and development

While the pace of basic biomedical research has been brisk, translating preclinical discoveries into meaningful clinical benefit using cancer immunotherapies has been difficult and mirrors the broader pharmaceutical industry [6]. Generally, drug discovery and development is a multi-phase process whereby basic knowledge of pathophysiology obtained through academic discoveries is translated into a medical entity that can be used to improve human health. The collective costs of bringing a therapy to the clinic reflect costs associated with acquiring data to support further development of a drug candidate but also include the costs associated with drug candidates that fail [7]. The high costs associated with drug development also contribute to increase patient costs. Eleven of 12 new cancer drugs approved by the FDA in 2012 cost greater than $100 K a year with ipilimumab leading the way at $250 K a year [8]. In recent years, phase II clinical trials have become a critical pinch point in the research and development pipeline, where the probability of success is lowest and associated costs are high [9-11]. The objective of Phase II is to demonstrate proof-of-principle in patients with the targeted disease.

The high risk of failure in phase II reinforces a herd mentality. Once a new mechanisms of action is found and a “first in class” drug comes to market, there is strong incentive for follow-on companies to develop similar drugs that target the same mechanism through a different means, with the objective to become “best in class” [12]. The current focus on immune checkpoint modulators illustrates this market phenomenon [13]. Clinical trials with an antibody targeting CTLA4 demonstrate the clinical proof-of-principle that immune checkpoint modulation can prolong survival for metastatic melanoma in a subset of patients [14, 15]. Follow on studies that inhibit the programmed cell death 1 pathway [16, 17], another immune checkpoint, suggest better efficacy and reduced side effects over targeting CTLA4 [18]. While a fast follower strategy helps mitigate the overall risk associated with the drug development process, the entry of many different companies that aim to be fast followers can fragment a market and narrow the window in which a “first in class” drug has sufficient market share to achieve a viable return on investment. Increased competition with “fast followers” is another factor in escalating drug prices, as the average time to market entry for fast followers has decreased [19]. Although entry of “fast followers” into the market can reduce patient costs through some price competition, drug prices can dramatically decline once generic drugs enter the market. The entry of generic drug competitors ends the window in which developers of either “first in class” or “fast followers” can recoup their increasing R&D investment [20]. The return on R&D investment is important as, from 2001 to 2014, half a trillion U.S. dollars in overall value of the top-tier pharmaceutical companies disappeared as the investment community shifted towards other more lucrative economic sectors^*a*^. Moreover, only 10 % of the drug companies that existed 50 years ago are still in business where the rest have either failed, divested, merged or been acquired [21]. Given this competitive landscape, sustaining the current investment to broaden the clinical impact of cancer immunotherapy across the patient spectrum will rely on continuously identifying new mechanisms of action [22].

### Invigorating drug discovery and development using quantitative and systems pharmacology

**Fig. 1:**
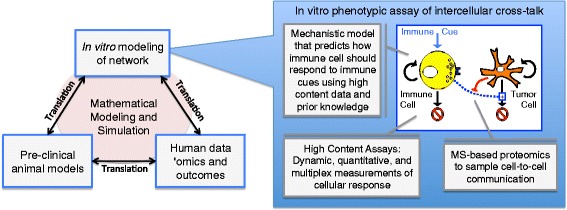
A summary of a quantitative and systems pharmacology approach to identify targets and to improve confidence in the selected targets. An integrated approach for cancer immunology combines in vitro modeling of cellular networks, pre-clinical mouse models of immune-mediated control of tumor growth, and human ’omics and clinical outcomes data from relevant patient populations. Mathematical modeling and simulation aids translational studies by representing the knowledge associated with the relevant biology and by testing this knowledge against data. The box on the right illustrates an example of in vitro modeling of an intercellular network, which is an in vitro phenotypic assay of intercellular cross-talk. The assay incorporates three quantitative and systems pharmacology aspects. First, the assay includes dynamic, quantitative, and multiplex measurements of cellular response. Second, this high content data is interpreted using a mechanistic mathematical model that incorporates prior knowledge about the relevant cell signaling pathways to predict how the immune cell should respond to immune cues, like Interleukin-12. Finally, a mass spectrometry (MS)-based proteomics workflow can be incorporated to characterize secreted cues that influence cell-to-cell communication. The targets that emerge from this integrated process and their associated “wet” and “dry” model systems can provide the rationale for screening for drug candidates using high throughput methods

In 2011, the National Institutes of Health organized an industrial and academic working group to study the challenges associated with drug discovery and development. The working group concluded that the lack of demonstrable efficacy in phase II clinical trials stem from an incomplete understanding of the biological mechanisms in humans that are targeted by the potential drugs [10, 23]. To address this bottleneck within the pipeline, this group coined a new field called quantitative and systems pharmacology (QSP) and drafted recommendations to reconnect pre-clinical drug discovery and development with human pathophysiology. There are two central themes associated with this new discipline. The first theme is the focus on a network-centric view of biology as a contrast to a “one-gene, one-receptor, one-mechanism” paradigm for drug discovery and development. The second theme of QSP is to integrate mechanistic modeling and simulation (i.e., “dry”-lab studies) with quantitative wet-lab studies. In the following paragraphs and summarized in Fig. 1, I will discuss these two themes and related concepts in more detail, with a particular focus on their relevance for the development of cancer immunotherapies.

#### Focus on a network-centric view of biology

**Fig. 2:**
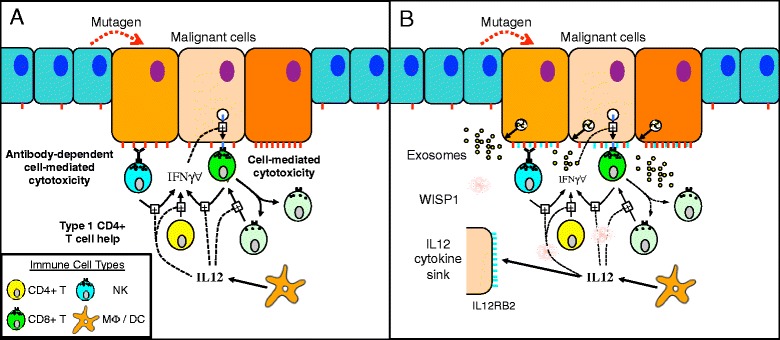
Interleukin-12 plays multiple roles within the local tumor microenvironment to sustain an anti-tumor response but malignant cells evolve mechanisms to suppress the local activity of IL12. **a** IL12 is secreted by macrophages and dendritic cells and acts on Natural Killer (NK) cells, CD4+ T helper cells, and CD8+ cytotoxic T cells to promote an anti-tumor immune response by enhancing antibody-dependent cell-mediated cytotoxicity and cytotoxic T cell activity. Effective anti-tumor immunity is also associated with local proliferation of CD8+ T cells within the tumor microenvironment [113]. Immune cell proliferation also diminishes the cellular response imprinted by IL12 stimulation [48], which suggests that local IL12 may help sustain the cytotoxic activity of CD8+ T cells. **b** As a result of somatic evolution, the B16F0 model for melanoma has evolved multiple mechanisms to suppress the bioactivity of IL12. B16F0 cells over express one component of the IL12 receptor, IL12RB2, to form a local cytokine sink (light blue whiskers on malignant cells) B16F0 cells secrete WISP1 (red cloud), which is a paracrine inhibitor of IL12 bioactivity, and exosomes (yellow circles). B16F0 exosomes contain IL12RB2, which can contribute to the cytokine sink, and deliver an immunosuppressive payload to suppress the proliferation of CD8+ T cells. As B16F0 exosomes are around 160 nm in size, they are likely to accumulate within the local tumor microenvironment

One of the main themes of QSP is the focus on a network-centric view of biology as a contrast to a “one-gene, one-receptor, one-mechanism” paradigm for drug discovery and development. This implies that instead of focusing on how well a drug modifies a specific molecular target in isolation, an emerging view is that drugs are best understood by focusing on how they modify the relevant biological network. Biological networks are commonly conceptualized at different scales within biological systems and depicted using nodes and edges (see Figures 2 and 5 in [24]). Intracellular signaling networks represent the spatially and temporally organized interactions between signaling proteins that enable the transport and storage of information within a cell to orchestrate a specific cellular response, like cytokine production or initiation of cell proliferation. Similarly, cells within a tissue communicate through biological networks. Based on a variety of experimental tools, our knowledge of the many of the molecular players within these intra- and intercellular signaling networks are known. However, identifying how these molecular players dynamically organize to control cellular response has been recently suggested to be a contemporary challenge in understanding how extracellular cues regulate cellular responses [25]. Moreover, the importance of specific nodes and edges can be vastly different among different tissues, genetic backgrounds, and stages of development or disease.

This leads to another recommendation from the QSP white paper, which is to reconnect tissue physiology with pharmacological testing using phenotypic screening platforms that are based on more complex systems. The essence of a phenotypic screening assay is that it recreates the relevant biological network in an experimental setting. Using this experimental system, biochemical cues can be tested for their ability to alter the network behavior, as represented by a change in phenotype [26]. This is in contrast to high throughput screening (HTS) assays, which primarily focus on engineering a reporter cell line to exhibit a defined response, such as GFP expression, when the biological activity of a particular protein target is modified using a drug [27]. A HTS system enables the rapid screening of chemical libraries for biological activity. Ideally, these two different screening approaches complement each other [23]. A phenotypic screening assay can be used to identify new targets. Once a target is identified and validated independently, a HTS assay can be used to identify potential drug candidates from large chemical libraries and prioritize these drug candidates based on their ability to modify the biology by interacting with the defined target. While conventionally this screening is performed in vitro, in vivo assays can also be used to screen libraries of shRNA candidates that target genes known to influence a specific biological function, such as the suppression of T cell function [28]. While the current emphasis within the industry is on HTS assays, phenotypic screening produced greater than 60 % of first-in-class small molecule drugs approved by the FDA between 1999 and 2008 [29]. Given that many biological networks involve dynamic and non-linear relationships, understanding how these networks work and how they become altered in disease from experimental observations is aided by recent advances in mechanistic modeling and simulation, which I will discuss in the next section.

#### Integrate mechanistic modeling and simulation with quantitative wet-lab studies

With the exponential increase in computational power, computer-aided modeling and simulation has transformed industries ranging from financial portfolio management to the aerospace industry. While these examples represent extremes, the role of mathematical modeling and simulation in these two instances are the same: it provides a quantitative framework to capture our conceptual understanding of the modeled process, interpret heterogeneous data acquired from the process and predict future behavior. Similarly, the goal of modeling and simulation in the context of QSP is to help bridge the innovation gap by providing a quantitative mechanistic framework to interpret clinical data by integrating quantitative, dynamic, and heterogeneous data obtained from in vitro studies and in vivo studies using animal models. In the following paragraphs, I will describe two examples where modeling and simulation were integrated with wet experimental studies to better understand how host immunity can be altered in disease states and to identify and prioritize therapeutic targets.

#### The Entelos PhysioLab platforms: virtual flight simulators for drugs

In the mid-1990, a number of companies formed that applied modeling and simulation technology to improve the drug development process, including Entelos and Physiome Sciences. At Entelos, PhD-level engineers and life scientists formed inter-disciplinary teams to develop mathematical models of disease, called PhysioLabs, that focused on specific disease areas within a proprietary modeling platform (e.g., [30, 31]). Simply stated, these mathematical models were a form of flight simulators for drugs. The models used math to connect receptor-level interactions with a clinical read-outs, such as blood glucose levels for diabetes or lung function measures for asthma, and were used to simulate the clinical response to existing and proposed therapeutics. A relevant example for cancer immunotherapy was the development of a PhysioLab that focused on type 1 diabetes. Type 1 diabetes is an auto-immune disease where the host immune system attacks the beta-cells present within the endocrine pancreas, which produce insulin to maintain glucose homeostasis [32]. Auto-immunity and cancer can be thought of as two different pathogenic consequences of a dysregulated host immune response [33, 34]. To better understand the factors associated with auto-immune diabetes, Entelos and the American Diabetes Association teamed up to create a PhysioLab based on type 1 diabetes. Considering the uncertainty associated with disease progression in humans (e.g., [35, 36]), the focus turned towards developing a PhysioLab platform for type 1 diabetes (T1D) based around the NOD mouse model [37]. The T1D PhysioLab incorporated immune effector activity in the endocrine pancreas, a secondary lymphoid organ that organizes the host immune response, and the trafficking of cells between these two locations [38]. In consultation with a scientific advisory board and using published experimental data, an interdisciplinary team of three PhD-level engineers and three PhD-level immunologists worked for two years to create this initial T1D PhysioLab platform.

The T1D PhysioLab was made available to the scientific community to share, as a clear cube^*b*^, the understanding of the underlying biology as represented by the model and to evaluate the predictive power of the approach. A number of published studies describe how such a platform can be used to prioritize immunotherapy targets [39-41]. For instance, one of the therapies currently under consideration is to tolerize patients to epitopes derived from insulin, which has been realized as a nasal insulin B:9-23 peptide therapy in the NOD mouse model. However one of the challenges for implementing such a strategy is the variety of values for treatment variables that could be selected, including the impact of dose, frequency of administration, and stage of disease progression. Understanding how these variables influence tolerance induction and the corresponding mechanistic prediction of the strength and dynamics of the immune response may help clarify conflicting reports on the efficacy of nasal B:9-23 peptide immunization in the NOD mouse. Moreover, these data may aid in translating antigen-specific therapies to humans, which has been difficult. Overall, the simulation results suggest that immunization frequency and the stage of disease were the primary variables [40]. Interestingly, low-frequency immunization increased Treg and IL-10 induction within the pancreas and protected animals from diabetes whereas a high frequency approach failed. These simulation results were subsequently confirmed using wet-lab experiments. One could envision a similar approach to evaluate ways to break tolerance to epitopes derived from tumor antigens [42].

The Entelos PhysioLab platforms illustrate how modeling and simulation can be used to address many of the hurdles associated with drug development [43]. In collaboration with industrial partners, these PhysioLab platforms were used to prioritize among competing targets, to explore the clinical implications of patient heterogeneity, and to assess the safety of a phase I clinical trial protocol (e.g., [31, 43-45]). In contrast to correlative methods to identify biomarkers (e.g., [46]), the PhysioLab platforms were also used to develop mechanism-based biomarkers for patient stratification or for use as early predictors of clinical efficacy, as a way to salvage a compound that appeared to fail in a phase II clinical trial for lack of efficacy. However, creating these models from scratch depends on pre-existing knowledge about what are the key elements within a specific network and the quantitative relationships among these elements, as inferred from the data extracted from the published literature. Creating these PhysioLab platforms also assumed that the physiology modeled could be captured using a modular approach. A modular approach is where smaller scale models are developed based on isolated components, such as a cell or signaling pathway (e.g., [47, 48]). These isolated components are connected together to capture higher level behavior, that is intercellular level responses, and calibrated using wet-lab observations of higher level behavior in response to lower level experimental manipulation. Examples of these wet-lab studies for the T1D PhysioLab include observing diabetes incidence in the NOD mouse following the antibody-mediated depletion of CD8 T cells. This approach has been described as a phenotype-driven modeling approach [43]. The key assumption in this approach is that data informing different aspects of the system must be obtained from the same system, that is the intracellular signaling-, cellular-, or tissue-level phenotypes observed must be self-consistent.

#### An integrated phenotypic screening approach for cancer immunotherapy

Recent genomic sequencing studies of cancer reinforce the idea that cancer arises through repeated rounds of mutation and selection, that is it is a process of somatic evolution [49-51]. Thus, cancer presents at least two challenges to this modular approach to modeling physiology. First, the heterogeneity of malignant cells either within a given tumor microenvironment or among tumors that arise in different patients presents challenges for obtaining self-consistent data across scales. Second, somatic evolution implies that cancer arises when the network of cellular communication within specific tissues have been altered to favor malignant cell survival. In the Biology of Cancer, Robert Weinberg states that cancer cells and normal cells “utilize control circuitry that is almost identical. Cancer cells discover ways of making relatively minor modifications of the control machinery operating inside cells. They tweak existing controls …” (pg 159) [52]. While the focus of Weinberg’s comment is on intracellular signaling networks, these changes also occur within intercellular signaling networks, as illustrated by a recent secretome comparison between normal human mammary and HER2+ breast cancer cell lines [53]. While a small study, the secretome analysis suggests that the particular proteins secreted by the malignant cells reflect a convergent evolutionary path associated with oncogenesis.

While there has been a number of factors identified within tumors that exert immunosuppressive effects [54-56], identifying the importance of these different mechanisms in specific patient groups remains a key hurdle for expanding the clinical benefit of cancer immunotherapy across the patient spectrum. As tumor immunosurveillance should be viewed as a integrated system [57], the role of specific elements within the tissue-level network may change with time and disease severity and also may vary in different anatomical locations. One approach to identify key control elements within the network of intercellular communication is examine the phenotype of genetically modified animals. Changes in cancer susceptibility in various knock-out mice suggest nodes within intercellular communication networks that may play important roles in immunosurveillance within specific tissues (e.g., [58-61]). Given that the biological roles of exosomes in normal and diseased tissues are only recently being revealed and remain controversial, there may be other secreted oncogenic factors that inhibit network nodes known to be important in tumor immunosurveillance and that act independent from immune checkpoints [62]. As part of an integrated strategy (see Fig. 1), phenotypic screening assays can be used to identify new mechanisms that tumor cells use to suppress these key regulators of tumor immunosurveillance. In the next section, I will describe a QSP-inspired phenotypic screening approach that focuses on identifying mechanisms that exhibit cross-talk with the cytokine Interleukin-12.

#### A QSP-inspired case study: Identifying local network re-wiring associated with Interleukin-12

As summarized in Fig. 2A, Interleukin-12 (IL12) is a type 1 cytokine that is produced by antigen presenting cells, such as macrophages and CD1c + Dendritic Cells, and acts upon Natural Killer (NK) cells, CD8^+^ Cytotoxic T cells, and CD4^+^ T helper cells [63]. Originally called Natural Killer cell stimulating factor, IL12 promotes the cytotoxic activity of NK cells and CD8^+^ T cells and promotes polarization of CD4^+^ T cells towards a type 1 phenotype. Interestingly, human CD4^+^ and CD8^+^ T cells introduced into the xenogeneic environment of humanized mice without IL12 preferentially differentiate into type 2 (IL4+ GATA3+) or mixed type 1 and 2 (IFNG+ TBET+ IL4+ GATA3+) subsets [64]. Injection of recombinant human IL12 into the mice was able restore differentiation towards a type 1 to imrove cytotoxic immunity to a viral challenge. In humans, genetic mutations in IL12p40 and one component of the IL12 receptor, IL12RB1, have been observed in patients with recurrent mycobacterial disease, suggestive of insufficient type 1 cell-mediated immunity [65, 66]. In mice, genetic deletion of other component of the IL12 receptor, IL12RB2, increases susceptibility to spontaneous autoimmunity, B-cell malignancies, and lung carcinomas [61].

As a drug, IL12 has been prioritized by NCI as one of the top four immunotherapies for cancer [67]. As a single agent, intravenous injection of recombinant IL12 exhibited modest clinical efficacy in a handful of patients with advanced melanoma and renal cell carcinoma [68, 69]. However, one death due to Clostridia perfringens septicemia in the first Phase I study limits interest in the systemic delivery of IL12 [70]. As a combination therapy, IL12 has been used as an adjuvant to enhance cytotoxic immunity using a melanoma antigen vaccine [71] or using peptide-pulsed peripheral blood mononuclear cells [72] and to promote NK-cell mediated killing of HER2-positive breast cancer cells in patients treated with trastuzumab [73]. Locally, delivery of IL12 to the tumor microenvironment promotes tumor regression in the B16 melanoma model [74], in the EL4 thymoma model [75], and in mouse models of glioblastoma in combination with CTLA4 blockade [76]. Interestingly, Kerkar et al. showed that IL12 within the tumor microenvironment acts on stromal cells, including macrophages and dendritic cells, to promote tumor regression [77]. Macrophages can suppress dendritic cell production of IL12 through the production of IL10 [78]. Conversely, delivery of exogenous IL12 via immunogene therapy promotes a phenotypic shift from myeloid-derived suppressor cells towards myeloid dendritic cells that present antigen [79]. Various strategies have been employed to enhance local delivery in vivo, including packaging recombinant IL12 in multilamellar liposomes [80] or with chitosan [81], engineering antibody-cytokine conjugates [82, 83], conditional gene therapy [84], and co-packaging IL12 gene expression with an oncolytic adenovirus [85]. Collectively, these results suggest that IL12 may be an important control node within the local immunoregulatory network, as genetic defects in IL12 signaling increase cancer incidence and enhanced local delivery of IL12 promotes tumor regression.

Given the importance of IL12 in locally promoting anti-tumor immunity, we hypothesized that malignant cells alter the selective fitness landscape by locally inhibiting the biological response to IL12. To test this hypothesis, we used an in vitro system that included the co-culture of the B16F0 cell line, one of the most commonly used transplantable models for metastatic melanoma [86], and the 2D6 cell line, a model of type 1 T helper cells [87]. The in vitro co-culture assay was selected for two reasons. First, we used a cell line, the 2D6 model, that exhibited a well-characterized response to IL12, which we can express in the form of a multi-scale mathematical model with demonstrated predictive power [48]. Specifically, the 2D6 cell secretes both IFN*γ* and IL10 in response to IL12 stimulation and secretes TNF*α* through an autocrine mechanism. The cellular production of IFN*γ* and IL10 were dose-dependently proportional to the level of STAT4 phosphorylation. The 2D6 cell line also does not express a signaling competent T cell receptor, such that antigen recognition is not a confounding variable. Alternative antigen-specific screening assays can be developed to focus on cues that influence T cell recognition [88-90]. The mathematical model provides a quantitative framework to interpret the observed cellular response during the in vitro co-culture.^*c*^ In short, the 2D6 cell model provides a stable platform to identify factors that suppress the bioactivity of IL12 within a variety of transplantable tumor cell models. If the role of IL12 in regulating host immunity is similar in different anatomical tissues and that suppressing the activity of this network node is essential for oncogenic transformation, as suggested by the murine knock-out studies, then malignant cells that arise in these tissues will harbor residual mechanisms to suppress the bioactivity of this cytokine.

The second reason why an in vitro co-culture model was used was that we could employ an unbiased mass spectrometry (MS)-based proteomics workflow to identify biochemical cues that are secreted by tumor cells that exert paracrine action on the 2D6 cell model. Unbiased proteomic methods are an emerging approach to characterize intercellular communication. For instance, De Boeck et al. used a MS-based proteomic approach to identify differences in secreted proteins between cancer-associated fibroblasts and non-cancer-activated bone marrow-derived mesenchymal stem cells that relate to colon cancer progression [91]. One of the challenges with this proteomics approach is that a relatively large sample size is required to identify secreted proteins by mass spectrometry and to validate using independent methods [92]. A reverse protein array, as described in [93], or possibly multispectral imaging of immunohistochemically labeled tumor tissue, as described in [94, 95], could be used as alternatives. However, these antibody-based approaches assume that the proteins responsible for the observed behavior are able to be quantified using an antibody and the selection of the specific proteins to observe is made a priori. An unbiased proteomics approach using mass spectrometry enables identifying both well-characterized proteins and proteins that have unclear biological roles, such as exosomes and WNT1-inducible signaling protein 1 (WISP1) [53, 96]. As an alternative to secretome profiling, direct imaging of protein, lipid, and small molecule profiles in tumor tissues using mass spectrometry is an emerging approach that could be used for tumor tissues [97, 98]. The distribution of lipid and small molecule profiles can be obtained at a lateral resolution of 10 – 350 *μ*m [99, 100]. However, discriminating between extracellular and intracellular localization and identifying higher molecular weight proteins is difficult given the current technology, although improvements are likely [101]. For instance, discriminating exosomes, which are between 100 to 200 nm in diameter under physiologic conditions, from parental cells is not currently possible. Overall, the use of MS-based proteomics methods to probe intercellular communication in specific biological contexts is a powerful approach but remains under-employed [92].

**Fig. 3:**
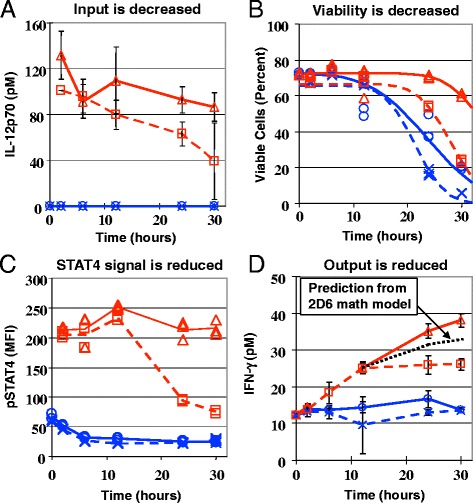
Evidence of cancer cell-immune cell cross-talk: functional inhibition of IL12 responsiveness in 2D6 cells following co-culture with B16F0 melanoma cells. The response of 2D6 cells to co-culture with B16 melanoma cells (dashed curves; □ + IL12; *×*-IL12) compared against 2D6 cells alone (solid curves; Δ + IL12; ○*-*IL12) in the presence (red curves) or absence (blue curves) of IL12, as reported in [96]. Co-culture of 2D6 with B16F0 cells reduced the level of IL12p70 (**a**) and viability of 2D6 cells (**b**). Following an induction period, pSTAT4 activity in (**c**) and the IFN-*γ* production by (**d**) 2D6 cells was inhibited by the presence of B16F0 cells. In panel D, the IFN-*γ* production, given the observed reduction in cell viability, was predicted using the mechanistic math model of 2D6 response to IL12 (dotted black line [48]). Changes in viability (**b**), IL12R*β*2 (not shown) and pSTAT4 in 2D6 cells (C - CD45+ events) were measured by flow cytometry. Conditioned media was assayed for IL12p70 (A), IFN-*γ* (D), TNF-*α* (not shown), IL-6 (not shown), and IL-10 (not shown) by cytometric bead array

As described in [96], a phenotypic screening assay was designed to monitor simultaneously multiple cellular parameters within a minimal biological system that recreates the desired phenotype. The cellular parameters assayed include 2D6 cell viability, 2D6 cell number, the intracellular signaling response to IL12, the level of IL12 in the cell culture media, and the cytokines produced by 2D6 cells that included IFN*γ*, IL10 and TNF*α*. Collectively, the different experimental measurements obtained at a number of different time points and experimental conditions provided 567 data points, which provide a high content view of the dynamic behavior of this assay. A number of changes were observed upon co-culture of the 2D6 cell with the B16F0 cell, as shown in Fig. 3. First, the levels of IL12 assayed in the conditioned media were consistently lower when the B16F0 cells were included in the co-culture. Second, viability of the 2D6 cells during prolonged in vitro culture times (*>*20 h) was improved by the presence of IL12 and diminished by the presence of B16F0 cells. STAT4, a key signal transducer in the IL12 signaling pathway, was phosphorylated in the presence of IL12, as expected. The level of STAT4 phosphorylation was the same for the first 12 h of IL12 stimulation, irrespective of whether B16F0 cells were present. At the 24 and 30 h time points, the level of STAT4 phosphorylation was decreased in viable 2D6 cells cultured in the presence of both IL12 and B16F0 cells. The decrease in STAT4 phosphorylation also corresponded to a cessation in IFN*γ* and IL10 production, which in the 2D6 model are dependent on IL12. The mathematical model was used to rule out the possibility that the observed reduction in IFN*γ* or IL10 was due to either the reduction in IL12 or in the viability of 2D6 cells in the presence of B16F0 cells. The delay in the observed cross-talk suggests that these inhibitory factors are not initially present within the system but require time to accumulate within the system. This would argue against inhibitory proteins expressed constitutively on the surface of B16F0 cells.

The delay associated with the appearance of immunosuppressive effects is a subtle but important aspect of tumor immunosuppression and is a common criticism of in vivo models that are used to test the efficacy of immunotherapies [102]. In this phenotypic screening assay, we recreate the dynamic appearance of paracrine immunosuppression. Assuming that the factors responsible for the observed immunosuppressive effects are constitutively secreted by the B16F0 cells, we used a proteomic workflow to identify these secreted proteins. We identified two secreted proteins, SPARC and WISP1, and a number of other proteins that are associated with exosomes [103, 104]. Collectively, the results from the phenotypic screening assay suggest that B16F0 cells use a variety of mechanisms to suppress the bioactivity of IL12 locally, as summarized in Fig. 2B. One of the mechanisms is the over expression of one component of the IL-12 receptor, IL12RB2, by B16 tumor cells and also by exosomes secreted by B16F0 cells, and suggests that these receptors create a local cytokine sink for IL12 [105]. Another one of the mechanisms used by B16F0 cells is the production of WISP1 that exerts a paracrine effect to suppress the bioactivity of IL12.

#### Infuse clinical and human ‘omics’-scale data into the pre-clinical discovery phase

Another recommendation from the QSP white paper is integrate diverse clinical and human ’omics’-scale data into the early stages of the drug discovery pipeline to help identify distinct patient populations and filter the potential therapeutic targets identified using the *in vitro* models of intercellular cross-talk for clinical relevance, as illustrated in Fig. 1. As the phenotypic screening assay identified a number of potentially novel local mechanisms of immunosuppression, I combined data obtained from the Cancer Genome Atlas with computer simulation to identify whether gene expression patterns consistent with the mechanism of immunosuppression identified in vitro are observed in human cancers and to identify particular patient subgroups where these mechanisms may be relevant [106, 107]. While many retrospective studies aim to discover biomarkers that are correlated with differential clinical outcomes (e.g., [108-110]), the goal here was to test whether the specific patterns discovered in our phenotypic screening assay were present in humans.

In the phenotypic screening assay, the first pattern that we observed was that both B16F0 and B16F10 over expressed one component of the IL12 receptor, IL12RB2. Over-expression of this receptor subunit and the corresponding reduction in IL12 in B16F0-conditioned media suggest that these malignant cells create a local cytokine sink for IL12. Using the TCGA data as a guide to select appropriate cell lines, differential expression of the components of the IL12 receptor is also observed on breast cancer cell lines that are associated with patient groups that exhibit enhanced anti-tumor immunity [106]. Collectively, these observations suggest that differential IL12 receptor expression is a remnant of immunoediting during somatic evolution of malignant cells and that this differential expression remains despite adaptation to in vitro culture.

**Fig. 4:**
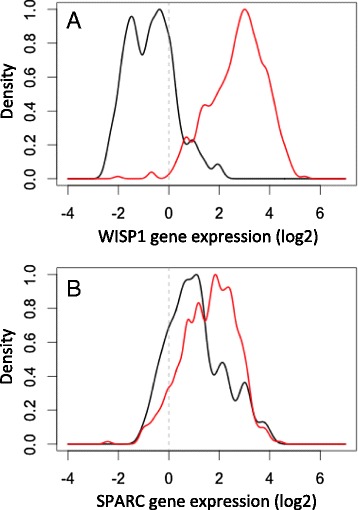
WISP1 is upregulated in tumor tissue samples obtained from patients with invasive breast cancer. The histograms for *WISP1* (**a**) and *SPARC* (**b**) gene expression assayed in homogenized tissue samples from invasive breast tumors (*n* = 520, red curves) and matched normal breast tissues (*n* = 61, black curves). Gene expression data were obtained from the invasive breast cancer arm of The Cancer Genome Atlas (TCGA) [116]

**Fig. 5:**
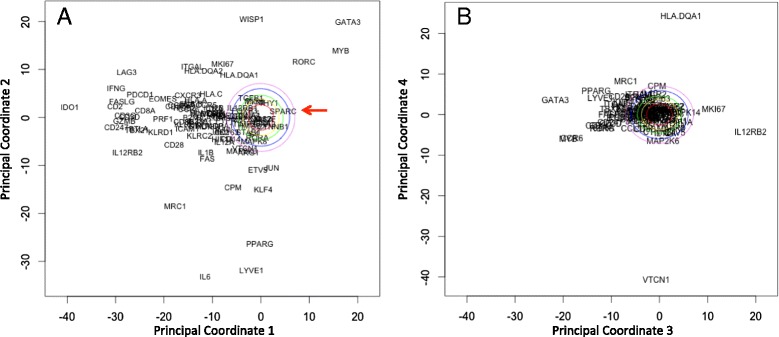
Increased *WISP1* expression correlates with increased *GATA3* expression. Principal coordinate analysis was applied to expression data for a subset of immune-related genes obtained from the invasive breast cancer arm of the Cancer Genome Atlas (see Figure S3 in [108]). Projections of the genes along the first two principal coordinate (PC) directions (**a**) and the third and fourth PC directions (**b**). PC 1 can be interpreted as a type 1 immune signature and PC 2 is interpreted as a signature of oncogenic transformation in invasive breast cancer. The first four principal coordinates capture 20, 13, 7, and 6 % of the overall variance in the data, respectively. As principal coordinates are independent, the projection of a gene along the corresponding axes indicates the degree to which the expression of two genes are related and the distance from the origin indicates the strength of the covariation within the data set. The remaining principal coordinates capture progressively less variance in the data and provide little additional information, as illustrated by the distribution of the genes along PC 4, except *HLA.DQA1* and *VTCN1*, are close to the origin. The colored ovals radiating out from the origin indicate principal coordinate values that can not be distinguished from random noise, that is a null hypothesis, with increasing levels of statistical stringency. The red arrow in panel A indicates *SPARC*. These colored ovals were obtained using a simulation approach called bootstrap resampling

The second pattern that we observed in the phenotypic screening assay was that tumor derived WISP1 has a paracrine effect to inhibit the bioactivity of IL12. In an initial survey, I observed that *WISP1* was upregulated in essentially all patients with invasive breast cancer, as shown in Fig. 4. In contrast, *SPARC* exhibited mixed results in patients with invasive breast cancer. As recently illustrated in vivo [64], the absence of IL12 skews T cell polarization towards a type 2 phenotype characterized by an increase in the transcription factor GATA3. Interestingly, one of the most pronounced gene expression signatures associated with *WISP1* expression was an up regulation in *GATA3* (see Fig. 5). These signatures were identified using principal coordinate analysis (PCA), which is a descriptive statistical technique used to find genes that exhibit coordinated expression patterns, and a focused analysis on immune-related genes, such as genes that define CD4^+^ and CD8^+^ T cells, NK cells, and alternative polarization states of T cells and macrophages. PCA allows for a lower dimensional representation of gene expression in terms of an individual genes expression within the entire data set attributed to each of the principal coordinates vectors. The genes with high loadings associated with a particular principal coordinate (PC) vector can be used to interpret a principal coordinate from a biological perspective. The PC vectors are ordered such that PC vector 1 captures the most variation in the gene expression data and additional PC vectors capture progressively less information. In this analysis, PC vectors 1 and 2 captured 33 % of the overall variance in the data.

However, the main question in this retrospective study is whether a gene expression pattern exists within the data that is consistent with our observation that WISP1 is a paracrine inhibitor of IL12. To address this question, an inferential statistics approach is required. While conventional hypothesis testing is difficult to do in this focused context, the central idea in hypothesis testing is to protect against the possibility that the observed effect can be explained by random noise associated with the experimental assay or the underlying biology [111]. By focusing on the particular genes associated with host immunity, I narrowed the universe of possible outcomes using prior knowledge about immune-related genes. To minimize concerns about cognitive biases, genes associated with pro-tumor in addition to anti-tumor host immunity and data from normal tissue samples were included in the analysis. From a hypothesis testing perspective, one can formulate the study question in terms of two hypotheses or models (*M*_*i*_): the null hypothesis (*M*_*o*_) that any identified relationship between *WISP1* and *GATA3* can be explained by random noise and the alternative hypothesis (*M*_*A*_) that the relationship between *WISP1* and *GATA3* is explained by a paracrine inhibition of IL12. Considering just these two alternative hypotheses, the inference problem can be expressed in a Bayesian framework:

1 $$ P\left({M}_A\Big|Y\right)=\frac{P\left(Y\Big|{M}_A\right)\cdot P\left({M}_A\right)}{P\left(Y\Big|{M}_A\right)\cdot P\left({M}_A\right)+P\left(Y\Big|{M}_O\right)\cdot P\left({M}_O\right)} $$

where *P* (*M*_*i*_*|Y* ) is the posterior probability in a particular model and *P* (*M*_*i*_) is the prior probability of the model.^*d*^ The likelihood of model *i*, *P* (*Y |M*_*i*_), can be expressed as the reciprocal of a comparison between an expected pattern (*Y*_*Mi*_) and the observed pattern of gene expression (*Y* ):

2 $$ P\left(Y\Big|{M}_i\right)\approx \frac{1}{{\left(Y-{Y}_{Mi}\right)}^2} $$

[113]. A gene expression pattern consistent with random noise can be constructed using computer simulation, that is by performing the analysis thousands of times on an equivalent synthetic data set constructed for each analysis by randomly sampling with replacement of the entire set of gene expression values. The potential PC projections of genes that could be explained by random noise are contained within the color ovals around the origin in Fig. 5. The negative correlation of *GATA3* with type 1-related immune genes in PC vector 1; which includes perforin, granzyme, *CD8*, and *IFNG* ; suggests that the strongest signal within the data set corresponds to a type 1 immune response. Moreover, the association of *GATA3* with the type 1 immune signature suggests that the *GATA3* signature is derived from immune cells and not epithelial differentiation. PC vector 2 captures the next largest co-expression signature in the data set, which includes a correlation between *WISP1* and *GATA3*. PC vector 3 captures an inverse correlation between *GATA3* and *IL12RB2*. Collectively, the first three PC vectors all provide gene expression patterns consistent with *WISP1* as a paracrine inhibitor of IL12 and that these patterns are very different from random noise. Assuming that the two hypotheses are equally plausible, the evidence supports the alternative hypothesis. We also note that *SPARC* appears within the colored ovals in Fig. 5, which suggests that any correlation between *SPARC* and immune polarization can be explained by random noise and argues against it’s potential as a therapeutic target. Finally, a pre-clinical mouse model can be used to further validate the role that these identified proteins play in immunosuppression.

Collectively, if these local mechanisms to suppress IL12 activity act similarly in humans, knowledge derived from these integrated wet and dry-lab studies could be used to stratify patients based on the prevalence of specific cross-talk mechanisms present within the tumor and guide selecting patient-specific immunotherapies. In a clinical trial setting, mechanism-based biomarkers that stratify patient populations could be used to improve the statistical power of less expensive clinical trials. In an adjuvant setting, mitigating these cross-talk mechanisms in conjunction with immune checkpoint modulators could expand the differential therapeutic window between productive local anti-tumor immunity and toxic peripheral effects due to auto-immunity, thereby broadening the clinical benefit of existing immunotherapies.

## Conclusion

While cancer immunotherapy is experiencing incredible clinical success, sustaining progress and maximizing the return to the community from the investment in human and financial capital requires a strategy for identifying new mechanisms of action. Despite a brisk pace of basic biomedical research, validating new mechanisms of action in humans has been identified as a key pinch point in the pharmaceutical research and development pipeline. Quantitative and systems pharmacology has been proposed as a new conceptual approach to address this challenge. Here, I have focused on a network-centric view of biology and the integration of mechanistic modeling and simulation with quantitative experimental studies as two central themes that help distinguish QSP as a new discipline. To illustrate the approach, two examples were chosen as they represent extremes of a spectrum of modeling complexity. The first example describes a hierarchical modeling approach where a large mechanistic mathematical model provided a framework to integrate a wide variety of experimental data, from in vitro observations to clinical trial results. Drawing from experiences using PhysioLab platforms, large-scale mechanistic models that integrate phenotypic signaling-, cellular-, and tissue-level behaviors have the potential to lower many of the translational hurdles associated with cancer immunotherapy. These include prioritizing immunotherapies, assessing the safety of proposed phase I clinical trials, developing mechanistic biomarkers that stratify patient populations and that reflect the underlying strength and dynamics of a protective host immune response, and sharing our understanding of the underlying biology using mechanistic models as clear cubes. Given the complexity of the models, they required significant resources to develop and rely on a modular approach to modeling physiology, which assumes that the biological networks remain similar in health and disease. To identify how cancer re-wires intercellular networks that locally regulate host immunity, the second example describes a flat modeling approach where the mechanistic models are more intimately connected to specific experimental data, which are acquired to inform the dynamic quantitative nature of the modeled biological system. As a consequence, the predictive power of the mechanistic model is more focused on a specific question but can be developed more rapidly. Here, this QSP-inspired phenotypic screening approach was used to identify local mechanisms that malignant cells use to suppress IL12 activity.

Overall, these two mechanistic modeling approaches each have their strengths and weaknesses in how they can help lower the translational hurdles associated with cancer immunotherapy. To paraphrase George E. P. Box [112], it is important to remember that, in some regards, all models are wrong but some may be useful for enabling one to think more clearly about the dynamic relationships among components of biological networks. While typically associated with mathematical modeling, this statement applies equally to biological and mathematical models. Inevitably, developing a model of a system involves abstraction, where key elements that are thought to be important in governing system behavior and their interactions are included while other components are left out to minimize confounding influences. The data are then used to inform the strength of the interactions included in the model. For instance, a common framework for modeling cell signaling networks is to assume that signaling events occur in the context of an average cell of constant volume. This framework neglects the impact of cell proliferation, which can reduce the concentration of activated species in the system through dilution alone. In such case, modeling interactions that regulate the activity of signaling proteins under conditions where cell proliferation may be important, such as during immune-mediated tumor regression [113], may lead to incorrect conclusions. Similarly, testing immunotherapies in mouse models prior to the establishment of immunosuppressive networks may lead to inappropriate inference as to the therapeutic potential of modulating a particular node [102]. Whether a component and its associated interactions are included or left out places conceptual boundaries on the specific questions that can be asked of a model. The value of model-based inference depends clearly on understanding these conceptual boundaries. The focus on mechanistic modeling within QSP is to use mathematical frameworks to be more explicit about these conceptual boundaries. The goal of QSP is then to facilitate a rational discussion about target prioritization and to use modeling and simulation methods to lower many of the translational hurdles associated with cancer immunotherapy.

## Endnotes

^*a*^The cumulative loss in market capitalization between Jan 2001 and May 2014 was $462 billion for the following companies: Pfizer, Merck, GlaxoSmithKline, Bristol-Myers Squibb, Lilly, AstraZeneca, Wyeth, Schering-Plough, and Abbott. Data from [114] and www.valueline.com.

^*b*^A clear cube is a simulation platform where all of the mathematical relationships are made explicit and are open to critique and review. The alternative is a black box, where the underlying mathematical details associated with a simulation platform are not made available for critique and review.

^*c*^While primary CD4^+^ and CD8^+^ T cells also respond to IL12, the intracellular signaling responses for cells that express the IL12 receptor, measured in terms of STAT4 phosphorylation by flow cytometry, exhibit a bimodal distributions [115] while the 2D6 cell line exhibits a unimodal distribution [48]. Changes in the distribution of cells between these two states independent from changes in signaling activation would complicate the interpretation of the data.

^*d*^Assuming that the proposed models are all equiprobable, the priors for the models cancel. Additional alternative hypotheses, such as WISP1 promotes T regulatory cell polarization or that SPARC is a paracrine inhibitor of IL12, can be included as part of the sum in the denominator.

## Abbreviations

HTS: High throughput screening
IL12: Interleukin-12
MS: Mass spectrometry
NK: Natural killer
PC: Principal coordinate
PCA: Principal coordinate analysis
QSP: Quantitative and systems pharmacology
R&D: Research and development
TCGA: The cancer genome atlas
T1D: Type 1 diabetes
